# Peer Review of “Waiting Time and Patient Satisfaction in a Subspecialty Eye Hospital Using a Mobile Data Collection Kit: Pre-Post Quality Improvement Intervention”

**DOI:** 10.2196/40448

**Published:** 2022-08-09

**Authors:** Haleh Ayatollahi

**Affiliations:** 1 Health Management and Economics Research Center Health Management Research Institute Iran University of Medical Sciences Tehran Iran; 2 Department of Health Information Management School of Health Management and Information Sciences Iran University of Medical Sciences Tehran Iran

**Keywords:** Waiting time, waiting list, patient satisfaction, quality improvement, clinical audit, ophthalmology, patient-centered care

*This is a peer-review report submitted for the paper “Waiting Time and Patient Satisfaction in a Subspecialty Eye Hospital Using a Mobile Data Collection Kit: Pre-Post Quality Improvement Intervention.”*

## Round 1 Review

### Reviewer BK

#### General Comments

In this paper [1], the authors aimed at improving patient waiting times and satisfaction through the use of PDSA quality improvement cycles. It is an interesting practical study. However, there are some major issues that need to be addressed by the authors. The following comments can help the authors improve the manuscript.

#### Specific Comments

##### Major Comments

1. In the Abstract and Methods sections, what does “ODK” stand for?
2. I suggest moving the problem description to the study rational as the first paragraph of this section.
3. In the methods section, the contents related to the data collection need to be expanded to include the type of data that were collected by data collectors.
4. The methods of data collection should be explained clearly.
5. In the Results section (page 11), the authors said, “The first 7 changes were implemented, which includes…” and “Five of the originally proposed changes could not be implemented due to…” I think it is better if the authors either change the wording of the sentences or provide a complete explanation of the all changes. Then, the authors can explain which strategy was implemented and which one was not implemented.
6. In the Results section (*Unintended Outcomes* subsection), the authors noted the following: “…the intervention appeared to have affected women adversely…” This section needs further explanations about the possible reasons for such an unintended outcome.
7. I am wondering whether all changes were implemented at the same time or they were implemented one by one. In case of the second approach, the impact of each strategy on changing waiting times and improving patient satisfaction could be investigated separately and compared with other strategies.
8. What were the possible reasons for non-significant increase in patient satisfaction while the waiting time was improved?
9. As the authors noted in the *Strengths and Limitations* section, the sample size was relatively small. However, they need to explain more why they did not reach a larger sample size. What were the main limitations?

##### Minor Comments

1. Multimedia appendices were not available to me.
2. Any survey instruments or questionnaires used for measuring patient satisfaction need to be added to the manuscript.

## Round 2 Review

### Reviewer BK

#### General Comments

I appreciate the authors for their time and efforts to implement our suggestions. However, some issues need further attention.

1. The Introduction section started with the problem description. This section usually comes later and after describing the background. Hence, the coherence of the paragraphs should be revised. Moreover, the current subheadings in the Introduction section seem unnecessary and the authors can remove or reduce them.
2. As the authors said, they implemented all the changes together. However, each strategy or change might have a different impact on changing waiting times and improving patient satisfaction, which was worth investigating. If the authors did not do so, it is better to add this point to the *Strengths and Limitations* section.
